# Distribution of HPV genotypes in cervical intraepithelial lesions and cervical cancer in Tanzanian women

**DOI:** 10.1186/1750-9378-6-20

**Published:** 2011-11-14

**Authors:** Adriana C Vidal, Susan K Murphy, Brenda Y Hernandez, Brandi Vasquez, John A Bartlett, Olola Oneko, Pendo Mlay, Joseph Obure, Francine Overcash, Jennifer S Smith, Mike van der Kolk, Cathrine Hoyo

**Affiliations:** 1 Department of Community and Family Medicine, and Program of Cancer Detection, Prevention and Control, Duke University School of Medicine, Durham, NC, USA; 2 Department of Obstetrics and Gynecology, Division of Gynecologic Oncology, Duke University School of Medicine, Durham, NC, USA; 3 University of Hawaii Cancer Center, Honolulu, HI, USA; 4 Department of Obstetrics and Gynecology, Duke University School of Medicine, Durham, NC, USA; 5 Division of Infectious Diseases, Department of Medicine and Duke Global Health Institute, Duke University School of Medicine, Durham, NC, USA; 6 Department of Obstetrics and Gynecology, Kilimanjaro Christian Medical Centre, Tumaini University, Moshi, Tanzania; 7 Department of Epidemiology, University of North Carolina, Chapel Hill, NC, USA; 8 Department of Medical Microbiology, Radboud University Nijmegen Medical Centre, Nijmegen, The Netherlands

**Keywords:** Human papillomavirus (HPV), cervical intraepithelial neoplasia (CIN), invasive cervical cancer (ICC)

## Abstract

**Background:**

Infection with human papillomavirus (HPV) is associated with uterine cervical intraepithelial neoplasia (CIN) and invasive cancers (ICC). Approximately 80% of ICC cases are diagnosed in under-developed countries. Vaccine development relies on knowledge of HPV genotypes characteristic of LSIL, HSIL and cancer; however, these genotypes remain poorly characterized in many African countries. To contribute to the characterization of HPV genotypes in Northeastern Tanzania, we recruited 215 women from the Reproductive Health Clinic at Kilimanjaro Christian Medical Centre. Cervical scrapes and biopsies were obtained for cytology and HPV DNA detection.

**Results:**

79 out of 215 (36.7%) enrolled participants tested positive for HPV DNA, with a large proportion being multiple infections (74%). The prevalence of HPV infection increased with lesion grade (14% in controls, 67% in CIN1 cases and 88% in CIN2-3). Among ICC cases, 89% had detectable HPV. Overall, 31 HPV genotypes were detected; the three most common HPV genotypes among ICC were HPV16, 35 and 45. In addition to these genotypes, co-infection with HPV18, 31, 33, 52, 58, 68 and 82 was found in 91% of ICC. Among women with CIN2-3, HPV53, 58 and 84/83 were the most common. HPV35, 45, 53/58/59 were the most common among CIN1 cases.

**Conclusions:**

In women with no evidence of cytological abnormalities, the most prevalent genotypes were HPV58 with HPV16, 35, 52, 66 and 73 occurring equally. Although numerical constraints limit inference, findings that 91% of ICC harbor only a small number of HPV genotypes suggests that prevention efforts including vaccine development or adjuvant screening should focus on these genotypes.

## Background

Globally, cervical cancer remains the third most common cancer, representing 8.8% of all cancers in women [1]. Infection with human papillomavirus (HPV) is associated with low-grade (LSIL) and high-grade (HSIL) intraepithelial precursor lesions of the uterine cervix as well as invasive cervical cancer (ICC) [2]. More than 200 HPV genotypes, subtypes and variants have been reported [3], of which approximately 14 genotypes are classified as oncogenic [4,5]. HR-HPV DNA is detected in almost all ICC cases [6].

In developed and in many less-developed countries, assessing the effectiveness of prophylactic vaccines against HR-HPV16 and 18, and further vaccine development will depend on knowledge of the distribution of these HPV genotypes in different regions [7-10]. HPV types 16 and 18 are consistently the two most common types in invasive cancer, globally. One study suggested that vaccination against HPV16 and 18 could prevent almost 70-80% of ICC worldwide-[11]. It has been suggested that vaccine efficacy could be increased to 95% for CIN2-3 and 92% for cancer by including an additional 12 HPV genotypes detected in an Icelandic women population [7]. However, data collected from different countries have shown fluctuations in the distribution of the third and even less common oncogenic HPV types between regions [9,12].

Given the suboptimal sensitivity and specificity of cytology-based screening, and the low screening coverage in most African countries, immunization against the most prevalent HR-HPV genotypes affecting each region may represent the most effective means to long-term ICC prevention [13]. To date, few studies describing single and multiple HPV infections according to CIN grades have been conducted in African populations where a wide range of HPV genotypes prevail [14-16]. Moreover, many of these studies have not described HPV infection in ICC cases [17,18]. Herein we report on the distribution of HPV genotypes in Northeastern Tanzania, with the aim of identifying the most frequent HPV genotypes associated with different CIN grades and ICC in this population.

## Methods

### Study participants

Procedures for this study were approved by Research Ethics Boards at KCMC and Duke University. Between November 2008 and March 2009, eligible study participants were identified from the appointment books of the Reproductive Health Clinic (RHC) at Kilimanjaro Christian Medical Centre (KCMC), a Cervical Cancer prevention clinic funded by the World Health Organization (http://www.afro.who.int/en/tanzania/who-country-office-tanzania.html). KCMC is a tertiary care facility that serves a catchment area of ~10 million individuals. Eligible participants were 18 years or older and had no history of an abnormal Pap test. ICC patients comprised new cases to the KCMC who were also 18 years or older and were referred for colposcopic directed evaluations. A trained nurse interviewer enrolled a total of 249 women; all but 2 approached agreed to participate (99% response rate). Of these, 12 patients were excluded due to missing or inadequate Pap smear, refusal of serostatus HIV-1 antibody test, and diagnosis of an unrelated co-morbid condition. The final dataset of participants, n = 215 (86%), were those with questionnaire, CIN status, and HPV genotype data.

### Data collection

#### Questionnaires

A trained nurse-interviewer obtained informed consent from all participants, and administered a standardized 40-minute questionnaire, in person. The questionnaire collected information on socio-demographic characteristics (e.g., age, marital status), type of marriage (polygamy vs. monogamy), tribe, educational attainment, cigarette smoking, alcohol intake, reproductive history (e.g., menarche, parity and gravidity), sexual history (e.g., lifetime number of sexual partners, age at first intercourse), and medication and supplement use.

#### Specimens

Two cervical scrapes were obtained from each participant using Ayres spatula and Cytobrush. One specimen was smeared on a glass slide for cytological evaluation for patient care. A second specimen was collected using a Cytobrush and rinsed into Preserv- Cyt™ media (Hologic, Inc, Malborough, MA). Following specimen collection, routine cervical screening by Visual Inspection with Acetic acid (VIA) was performed. Biopsies of lesions were obtained during colposcopy when indicated. Patients with positive findings by VIA or direct examination were triaged and treated accordingly. The remaining patients were given return appointments to follow up on results within two weeks, and were treated accordingly.

#### Ascertainment of CIN and Carcinoma

Papanicolaou smears and biopsy specimens were processed and read by the staff pathologist at KCMC- using standard conventions according to ASCCP guidelines as appropriate (http://www.asccp.org/). Once a month, medical charts were reviewed by BV for HIV-1 test and cyto-pathological results, to classify cases using the Bethesda classification system [19]. Based on pathology and medical records findings, results were then coded as "no evidence of cytological abnormality", "mild dysplasia" including LSIL and CIN1, "moderate dysplasia" including HSIL and CIN2-3, or "cancer" which included squamous cell carcinoma and two adeno-squamous carcinomas of the uterine cervix. None of the specimens were read as "atypical cells of uncertain significance (ASCUS)". These results were available as part of their clinic records, and pathologists entered them into the database. These clinical results were then compiled and transferred securely to Duke University.

#### HPV genotyping

ThinPrep^® ^specimens and homogenized aliquoted biopsies collected during the same visit were shipped to the University of Hawaii Cancer Center. Following DNA extraction, PGMY09/PGMY11 primers [20] were used in PCR to target a 450-bp region of the HPV L1 genome. Amplification of the human β-globin gene was included as an internal control for sample sufficiency. All specimens were suitable for viral DNA analysis. HPV-positive specimens were subsequently genotyped by using the HPV Linear Array^® ^(Roche Molecular Systems Inc., Branchburg, NJ, USA).

#### Ascertainment of HIV-1 infection status

Peripheral blood samples were centrifuged to separate the plasma and buffy coat. Plasma samples were used to test for HIV-1 infection using two rapid HIV tests (Capillus HIV-1/HIV-2, Trinity Biotech PLC, Bray, Country Wicklow, Ireland, and Determine HIV-1/2, Abbott Laboratories, Abbott Park, IL). Reactive specimens were then tested using Western blot as is standard clinical practice (Genetic Systems HIV-1 Western blot kit; Bio-Rad, Hercules, CA) [21].

### Statistical analyses

For each disease endpoint (CIN1, CIN2-3, ICC and women with no evidence of cervical abnormalities), we computed the proportion of single and multiple HPV infections, and grouped them according to potential oncogenicity using WHO-recommended categories [22]. Group 1 comprises high-risk (HR) or the most potent type HPV16, followed by HPV18, 31, 33, 35, 39, 45, 51, 52, 56, 58, and 59; group 2A includes HPV68; group 2B includes HPV26, 53, 66, 67, 70, 73, and 82 with limited evidence for human cervical cancer; and group 3 includes low-risk (LR) HPV types 6 and 11 [22]. We estimated the average number of HPV infections per woman by dividing the total number of HPV genotypes in multiple infections by the number of women infected. To estimate the attribution of each HPV genotype to CIN or ICC beyond the known HR-HPV, we estimated proportions in each case group with and without HR-HPV genotypes. Statistical analyses were conducted using SAS 9.2 (SAS Institute, Cary, NC).

## Results

Cervical cytology and HPV testing was conducted among 215 Northern Tanzanian women, 55% were Chaga, 15% were Pare and 30% were from other tribes. Although the median age of all cases was 45 years, women with no evidence of cervical abnormalities or controls (mean age 40.3 years, sd = 9.87), and CIN1 cases (mean age 35.7 years sd = 12.2) were younger than women with CIN2-3 and ICC whose mean ages were 44.7 years, (sd = 9.82) and 55.2 years, (sd = 12.3), respectively. Controls and case groups did not vary significantly by tribe, cigarette smoking and lifetime number of sexual partners, although controls were less likely to be infected with any HPV. HIV-1 infection was more frequently reported by CIN than ICC cases or controls while OC use was more frequently reported by CIN cases and controls than ICC cases (Table 1).

**Table 1:** Socio-demographic characteristics of study participants

	Cancer (n = 48)		CIN2-3 (n = 17)		CIN1 (n = 21)		CONTROLS (n = 148)		p-values**
	n	%	n	%	n	%	n	%	
**Age**	55.2	12.3	44.7	9.8	35.7	12.2	40.3	9.9	<0.0001
**Income $**	18	28	39	53	65	80	78	95	<0.0001***
									
**Lifetime sexual partner**									0.4* (1,2,3,4)
1	24	52.2	7	43.8	5	25	62	56.5	
2	19	41.3	6	37.5	11	55	56	39.2	
3	3	6.5	2	12.5	4	20	20	14	
4	0	0	1	6.3	0	0	2	1.4	
None	2		1		1		5		
									
**Marital status**									0.6*
Married	30	62.5	10	58.8	15	71.4	107	72.3	
Non-married	18	37.5	7	41.2	6	28.5	40	27	
									
**HPV**									<0.0001
Any	33	89.2	14	87.5	12	66.7	20	14.1	
None	4	10.8	2	12.5	6	33.3	122	85.9	
Genotypes per Case	2.8		3.7		2.7		2.1		
									
**Current Smoke**									0.6*
Yes	2	4.2	0	0	0	0	3	2	
No	46	95.8	17	100	21	100	144	98	
									
**Oral contraception use**									0.003
Yes	19	39.6	10	58.8	16	76.2	98	67.6	
No	29	60.4	7	41.2	5	23.8	47	32.4	
									
**HIV-1 infection**									<0.0001*
Yes	6	19.4	9	81.8	9	64.3	23	20.9	
No	25	50.6	2	18.2	5	35.7	87	79.1	
									
**Tribe**									0.3
Chagga	21	43.8	12	70.6	10	47.6	84	57.1	
Pare	10	20.8	3	17.7	2	9.5	21	14.3	
Others	17	35.4	2	11.8	9	42.9	42	28.6	

• * p-values is from fisher's exact chi-squared test due to small cell

• ** p-values are exclude missing

• *** Poisson regression

• Numbers do not necessarily add up to the total due to missing values

Table 2 shows the total number of HPV-infected individuals and the distribution of HPV genotypes by cervical intraepithelial lesion or cervical cancer status. Of 215 participants, 79 (36.7%) were HPV-positive and of these, 20 (25%) had no evidence of cytological abnormality, 12 (15%) had CIN1, 14 (17%) had CIN2-3, and 33 (41%) had cervical cancer. Among 134 HPV negative women, only 4 (3%) had cancer, 6 (4.5%) had CIN1, and 2 (1.5%) had CIN2-3. Overall, 31 distinct HPV genotypes were detected, and their detection rates, including co-infection with more than one genotype are illustrated in Table 2. The prevalence of HPV infection increased with lesion grade: 14% in controls, 67% in CIN1 cases, 88% in CIN2/3 and 89% in ICC.

**Table 2:** Distribution of HPV types in CIN1, CIN2, CIN3 and Cervical cancer

HPV test results	Cancer (n = 33) (%)		CIN2- 3 (n = 14) (%)		CIN1 (n = 12) (%)		Controls (n = 20) (%)	
	**Single**	**Multiple**	**Single**	**Multiple**	**Single**	**Multiple**	**Single**	**Multiple**
**High-risk HPV type**	n = 8	n = 70	n = 3	n = 28	n = 4	n = 22	n = 4	n = 29
Group 1								
16	62.5	72.7	33.3	14.2	0	16.6	16.6	15.0
18	0	21.2	33.3	14.2	0	0	16.6	10.0
31	0	18.1	0	14.2	0	0	0	5.0
33	0	9.09	0	14.2	0	0	0	0.0
35	25.0	33.3	0	14.2	25.0	25.0	0	15.0
39	0	0	0	0	0	8.3	0	0
45	12.5	30.3	0	7.14	0	25.0	16.6	10.0
51	0	0	33.3	14.2	25.0	16.6	0	10.0
52	0	15.1	0	0	0	8.3	0	15.0
56	0	0	0	7.14	0	0	0	5.0
58	0	6.06	0	28.5	50.0	25.0	33.3	25.0
59	0	0	0	7.14	0	25.0	0	5.0
**Low-risk HPV type**	n=0	n=17	n=0	n=8	n=0	n=7	n=3	n=10
Group 2A								
68	0	3.03	0	7.14	0	0	0	5.0
Group 2B								
26	0	0	0	0	0	0	33.3	5.0
53	0	0	0	35.7	0	25.0	0	5.0
66	0	0	1	14.2	0	0	33.3	15.0
70	0	0	0	0	0	8.3	0	10.0
73	0	15.1	0	21.4	0	8.3	0	15.0
82	0	3.03	0	7.14	0	8.3	33.3	10.0
Group 3								
6	0	33.3	0	7.14	0	0	0	0
11	0	9.09	0	0	0	8.3	0	0
**Other types**	n = 0	n = 6	n = 1	n = 16	n = 0	n = 4	n = 1	n = 3
40	0	0	0	0	0	0	0	0
42	0	0	0	7.14	0	8.3	100	5.0
55	0	3.03	0	7.14	0	16.6	0	5.0
61	0	0	0	7.14	0	8.3	0	5.0
62	0	3.03	0	21.4	0	16.6	0	0
69	0	0	0	14.2	0	0	0	0
72	0	0	0	7.4	0	8.3	0	5.0
81	0	6.06	0	21.4	0	0	0	10.0
83	0	0	0	28.5	0	16.6	0	0
84	0	0	100	28.5	0	0	0	0

### HPV genotypes in women with CIN1

HPV DNA was detected in 67% of CIN1, and 50% of the HPV detected were of HR genotypes. On average, there were approximately 3 HPV genotypes per CIN1 case (range 0-9). Sixty percent of women with CIN1 lesions had multiple infections. In order of prevalence, the seven most frequent HR-HPV genotypes detected among CIN1 cases were: 35, 45, 53, 58, 59, 16, and 51, followed by low risk (LR) type HPV55. In two individuals the rare HPV62 type was detected (Table 2).

### HPV genotypes in women with CIN2-3

HPV DNA was detected in 88% of CIN2-3; on average 4 HPV genotypes were detected per case (range, 0-9) as also found for women with CIN1. High-risk HPV subtypes were found either as single or multiple infections in 57% of CIN2-3 cases. In contrast to 73% of ICC which harbored the HPV16 genotype, only 3 (14%) CIN2-3 cases, 2 (17%) CIN1 cases and four controls (15%) had detectable HPV16 DNA. Multiple HPV infections were also common among CIN2-3 cases with 75% showing HPV coinfections. Also as in CIN1, 50% of the HPV genotypes detected were HR while the remainder were LR or other types of unknown oncogenic potential. HPV53, 58, 16, 18, 31, 33, and 35, were the seven most frequent types detected among women with CIN2-3, in order of decreasing frequency (Table 2). LR-HPV genotypes were detected in CIN1 and CIN2-3 lesions only in the context of multiple infections with HR genotypes.

### HPV genotypes in women with ICC

HPV DNA was detected in 89% of ICC biopsies with an average of 3 genotypes per case (range 0-6). High-risk HPV subtypes were found either as single or multiple infections in 78% of ICC. Most (~73%) of the ICC cases harbored the HPV16 genotype either as a single infection (15%) or along with other HR (30%) or LR (9.1%) genotypes. In contrast, HPV 16 was rare in women with lower CIN grades as only 2 of 14 (14%) CIN2-3, 2 of 12 (17%) CIN1, and 3 of 20 (15%) of women with no evidence of cytological abnormalities harbored HPV16 infections. Following HPV16, the next most frequent genotypes in ICC were HPV35, 45, 18, 31, 52, 33 and 58 (Table 2). These frequencies were all statistically significantly higher than in controls. Interestingly, HPV18 was found in 21% of ICC, and only when HPV16 was also present.

### Single versus Multiple HPV Infections

Table 3 shows the distribution of single and multiple HPV infections by lesion grade and cervical cancer status. HR-HPV 18, 35, 45 and 31, together with HPV16 were detected in 60.6% of ICC cases. HR-HPV genotypes 33, 52, 58, 68 and 82, in multiple infections with HPV 16, were detected in 30.3% of ICC cases. When combined, HR-HPV genotypes 16, 18, 31, 33, 35, 45, 52, 58, 68, and 82, they accounted for 90.9% of all HPV positive ICC cases. HPV16 multiple infections with LR-HPV genotypes 6, 11, 61, and 81 and other HPV genotypes (62 and 73), comprised the remaining 9.1% of HPV positive ICC cases. Among all CIN cases, HPV16 was not the prevalent genotype detected; 93% of CIN2-3 and all of CIN1 cases had multiple infections with HPV18, 26, 35, 45, 51, 53, 56, 58, 59, and 66 (Table 3). Whereas of 120 controls or women with no evidence of cytological or histological abnormalities, only 20 (14%) were HPV-positive, in which 90% of multiple infections we detected harbored the same genotypes (HPV18, 26, 35, 45, 51, 53, 56, 58, 59, and 66 (Table 3).

**Table 3:** Distribution of HPV16 and other most prevalent genotypes in CIN lesions and ICC

HPV genotypes	ICC Total (%) n = 33	CIN2-3 Total (%) n = 14	CIN-1 Total (%) n = 12	Controls Total (%) n = 20
HPV-16 only	5 (15.1)	1 (7.1)	0 (0.0)	1 (5.0)
HPV16 with HPV-18 only	5 (15.1)	2 (14.2)	0 (0.0)	2 (10.0)
HPV16 with HPV 18,35,45, or 31	20 (60.6)	2 (14.2)	1 (8.3)	3 (15.0)
HPV16 with HPV 18,31,33,35,45,52,58,68,82	30 (90.9)	2 (14.2)	1 (8.3)	3 (15.0)
HPV16 with HPV 6, 11,18,31,33,35,45,52,58,61,62,68,73,81,82	33 (100)	3 (21.3)	2 (16.6)	4 (20.0)
Other HR-HPV single infections 18,35,45,51,58,66	33 (100)	4 (28.6)	6 (50.0)	8 (40.0)
HPV 18,26,35,45,51,53,56,58,59,66, excluding HPV16	33 (100)	13 (92.8)	12 (100)	18 (90.0)
HPV 18,26,35,42,45,51,53,55,56,58,59,61,66,69,70,72,81,83,84, excluding HPV16	33 (100)	14 (100)	12 (100)	20 (100)

## Discussion

Our key finding was that among women visiting the KCMC, a tertiary care facility serving 10 million people, in Northern Tanzania, HPV16, 35 and 45 alone accounted for most of the invasive cervical cancers found. These HPV genotypes were rare in CIN lesions regardless of grade, suggesting that a vaccine that includes these three genotypes could prevent ~70% of ICC. Our findings also suggest that ICC risk is higher in individuals infected with multiple HPV genotypes, including those considered as high and low risk, suggesting that the cumulative burden of HPV multiple infections, maybe a marker of differential immune response. We did not detect any HPV DNA in 11% of ICC cases, a proportion within the 6-16% range of HPV-negativity in ICC previously found [11], and similar to the 11.5% reported elsewhere [8].

While findings of HPV16 association with ICC are consistent with -current knowledge, our data also suggest that in addition, HPV35 and HPV45 may increase risk, as observed in a global meta-analysis study [23]. This interpretation is consistent with data found in other African countries, where HPV16 and HPV18 were less common than other HR types in CIN1, though involved in more advanced lesions and cervical cancer [24-28]. Most of these studies used fresh biopsies for HPV detection [24,26,27]. We detected HR-HPV16 in 73% of ICC cases, including single and multiple infections. In the US, HPV16 and 18 combined are found in cervical cancer at a rate of 70.2% [23], similar to findings in a Canadian study: HPV16 DNA in 52.1% of ICC and HPV18 DNA in 18.1% of ICC [8]. However, the percentage of African women in the Canadian study was 3.9% and geographic regions of origin are unknown, while our population was 100% African [8,29]. If these results can be replicated in a larger study, our findings would suggest that the association of HPV16 and ICC in the Tanzanian region may be much higher than that in other populations. Also, we found five ICC cases with HPV16 single-genotype infection. However, there were no ICC cases with single-genotype HPV18 infection.

HPV 35/45/6 and 18/31/73 were the next most frequent genotypes detected in ICC, partly matching reports by others, where HPV31, 33 and 45 were among the six most frequently detected genotypes in ICC in North America [8-10,23]. Our results are similar to those of the Canadian study, where HPV45 was one of the three most prominent genotypes in ICC, ranking ahead of HPV31 and 33 [8]. We could not identify a clear trend for a specific HPV type that would suggest lesion progression into cervical cancer. However, HPV35 and 58 were the only types detected in CIN1, CIN2-3 and ICC. HR-HPV genotypes 16, 35 and 45 were the major genotypes found in cervical cancer cases.

Our results show some interesting differences with global cervical cancer data. The most frequent genotypes we detected in CIN1 were HPV35, 45 and 53/58/59, which differ from the most common types detected in CIN1 in the US, including HPV16, 66, 31, 52 and 51 [10], and HPV 16, 51, 52, 39, 18, 31 in Canada [8]. In CIN2-3 lesions, we predominantly detected HR-HPV 53 and 58, and the rare genotypes HPV83 and 84. These findings contrast with those suggesting that the most prevalent genotypes found in CIN2-3 in the US were HPV16, 31 and 18, however HPV58 ranked most frequently after 16, 31, and 18 [9,10,23,30]. Disparities among these studies may be due in part to different methodologies employed; however the evidence suggests that geographical differences contribute to HPV type distribution in cervical intraepithelial neoplasias across global regions, as reported [23].

The finding that HPV58 was the most prevalent genotype, followed by 16, 35, 52, 66, and 73 in equal proportions, are partly consistent with those of a recent meta-analysis in which the most common HPV genotypes among women with normal cytology in Eastern Africa are HPV52, 16, 18, 53 and 66, in order of prevalence [31]. In a study in Northwestern Tanzania [17] HPV16 and 33 were the main genotypes present in women with HSIL or advanced CIN; however this study had a small HSIL sample size (5 of 19 women) and no cancer cases. The most common genotypes detected among LSIL and HSIL cases were HPV16, 58, 33 and 18. In Mozambique, HPV35 was detected as the most prevalent type among HPV-positive women and among women with HSIL [14]. A Zimbabwean study found HPV16, 58, 18 and 52 to be the most common genotypes [32] in HIV-1 seropositive women with multiple HPV infections. But a European population-based study could not verify the oncogenic potential for HPV 58 and 59 in Icelandic women [7]. In our study, our rankings were not influenced by HIV-1 infection, (data not shown).

Findings that multiple HPV infections were more common than single infections in CIN1, CIN2-3 and cancer cases match reports by others [8,33-35]. While it has been shown that the risk for persistent infection with one HPV genotype is not synergistically enhanced by an existing infection with another genotype [11,36,37], others [38] suggest local levels of HPV-specific immunoglobulin G (IgG) and IgA in the infected tissue may be insufficient to clear viral infections [38]. Thus, factors that affect humoral immunity, such as genetic predisposition, frequency of re-infection, genetic variations of the HPV genotype, and hormone levels could explain, at least in part, the association between multiple HPV infections and CIN and ICC in this region. When adjusting for HIV-1 infections in HPV-positive women, we found 50% more CIN1 and CIN2-3 lesions in HIV-1 seropositive women with HPV multiple infections (65%), than in HIV-1 seronegative ones (30%). While this would support the hypothesis that immunocompromised individuals are at higher risk of acquiring multiple infections, 84% of the ICC cases here reported were HIV-1 negative individuals, (data not shown).

The main limitation of our study is that these analyses relied on a small number of cases. However, findings contribute to HPV knowledge in East Africa that will be useful for vaccine development and to augment screening strategies. East Africa continues to have one of the highest ICC incidence and mortality [39], and cytology-based screening coverage remains low. Another limitation is that HPV DNA was detected from exfoliated cells for CIN cases and controls as biopsies were not medically indicated. Thus, some of the HPVs detected may not have been related to lesions subsequently detected. However, cells from homogenized biopsy specimens were used for HPV detection in the all squamous cell ICC cases. Therefore, limitations notwithstanding, our ICC findings support the hypothesis that the cumulative burden of multiple HPV genotypes may contribute to ICC incidence, presumably via altered immune response.

## Conclusions

We found that HPV 16, 35 and 45, but not HPV18, were the most common HPV subtypes in ICC. HR-HPV genotypes 45, 53 and 58 significantly contribute to CIN1, CIN2-3 and ICC rates in Northern Tanzanian women. Multiple infections were found in the majority of cancers and high grade CIN. Our data suggest that a vaccine targeting HPV genotypes 16, 18, 31, 35 and 45, could help prevent up to 61% of ICC in this region; while adding HPV genotypes 33, 52, 58, 68, and 82 may help increase prevention up to 91% of ICC. The low frequency or absence of HPV 16 and 18 in CIN1 or CIN2-3 may have implications for promoting cytology and HPV-based screening in this region. Larger studies are required to confirm these findings.

## Competing interests

The authors declare that they have no competing interests.

## Authors' contributions

AV analyzed the data, run the statistical analysis and drafted the manuscript. SKM participated in the design of the study and in the drafting of the manuscript. BYH carried out the HPV genotyping. BV participated in the study design and coordination. JAB, OO, PM and JO actively participated in the overseeing of the recruitment, sample collection, and study design. FO managed the data. JSM and MVDK participated in the drafting of the manuscript. CH conceived of the study, participated in its design, coordination, and data analysis and participated in drafting the manuscript. 

All authors read and approved the final manuscript.
